# Dr. SB Wey: a path of influence and discovery in hospital epidemiology

**DOI:** 10.1017/ash.2024.440

**Published:** 2024-10-17

**Authors:** Sergio B. Wey

**Affiliations:** Hospital Israelita Albert Einstein, São Paulo, Brazil

## Introduction

Dr Sergio Barsanti Wey is a professor at the Escola Paulista de Medicina/ Universidade Federal de São Paulo (EPM/UNIFESP), in São Paulo, Brazil. Dr Wey initiated research in Hospital Epidemiology within the Infectious Diseases Discipline upon returning from a Fellowship program at the University of Iowa in 1987. He was driven to pursue studies in the United States with the mission of establishing a hospital infection unit at the (EPM/UNIFESP), as at that time in the 1980s, infection control units were embryonic in Brazil. Since then, his primary research focus has been on the Epidemiology of Hospital Infections. In addition to supervising numerous master’s and doctoral students, he co-coordinates the postgraduate course in Hospital Infection Control. In 2020, like several epidemiologists, Dr Wey served as a consultant to various medical and non-medical sectors on issues related to COVID-19. Among these, he advised the CBF (Confederação Brasileira de Futebol), the Brazilian Football Confederation on safely resuming football matches for players and spectators alike. Now more connected to clinical practice, he continues to participate in the development of research and teaching in hospital epidemiology for young health professionals. His current projects involve machine learning and artificial intelligence at his institution, the Hospital Israelita Albert Einstein in São Paulo, Brazil.

## Tell us about your unique training path that led you to infection prevention?

I left Brazil to establish an infection control center for EPM/UNIFESP. All of this was caused by one of the Brazil’s presidents, Tancredo Neves, who was elected through the “Diretas Ja” movement, after the end of the military dictatorship. He fell ill a few days after taking office and died of sepsis due to a peritonitis acquired from hospital infection a few weeks later. This tragic event caused a national commotion and motivated the creation of infection control units around the country (Brazil) to prevent similar incidents in the future.

The hospital administration affiliated with EPM/UNIFESP contacted the Centers for Disease Control and Prevention (CDC), which recommended I undergo training with Dr Richard Wenzel in Charlottesville, Virginia. I had the honor of being the first international fellow from Brazil of Dr Wenzel. Telling the story from Charlottesville, VA to Iowa: At that time, there was no internet, no Global Positioning System (GPS), etc. Communication was difficult. There were no computers, no PubMed, etc. I started with practical training on hospital surveillance as a model, in Charlottesville, VA, always under the guidance of Dr Wenzel. Then, when Dr Wenzel was invited to go to Iowa City, Iowa, I followed him there as well. In Iowa, I aimed to focus more on research. I went to learn a method that, for most of you, might seem trivial now: learning to evaluate risk factors for candidemia using the case-control study design. All of this was Dr Wenzel’s idea.

To illustrate, Dr Wenzel invited two professors to Iowa to evaluate a series of projects, including mine on candidemia. These professors were Dr Alvan Feinstein and Dr Robert Fletcher, renowned epidemiologists with extensive experience in population studies, and Dr Wenzel wanted to know if this type of study, namely case-control, would be valid within a hospital setting. They said yes, and that’s how I was able to conduct my first research using this design, which has become so popular in our field of hospital epidemiology.

Dr Wenzel was unsure if *Candida* could be an indicator of someone who might die or a risk factor significant enough to intervene. This led us to work on two projects: risk factors for candidemia^1^ and risk factors for mortality attributed to *Candida*.^2^ These two studies were crucial for my academic career because by validating this case-control study method in a hospital, I was able to replicate other studies in Brazil and also teach other students and researchers interested in epidemiology in my country and Latin America as well.

Additionally, these studies gave me significant visibility as a researcher, along with Dr Wenzel’s generosity in inviting me to write a book chapter on candidemia in “Prevention and Control of Nosocomial Infections.” These stories are amusing. How did I manage to write without a computer or PubMed at the time? To conduct the book review, I had to stay for three weeks in North Carolina, where Dr William Rutala kindly allowed me to use the university library.

Dr Wenzel also invited me to join the editorial board of ICHE, which at that time was called the “Infection Control” journal until it was renamed Infection Control and Hospital Epidemiology. Those two years were short, but undoubtedly among the most important of my life, marking a turning point. I always think of those moments with great affection.

Moreover, medical conferences were very important to me. I made several contacts with other epidemiologists at Society for Healthcare Epidemiology of America (SHEA) and Interscience Conference on Antimicrobial Agents and Chemotherapy (ICAAC). Unfortunately, ICAAC ended, but now we have IDWeek and SHEA still alive.

## Describe a pivotal mentor relationship that altered the trajectory of your career

As I mentioned earlier, Dr Wenzel was, without a doubt, incredibly important to me. You can already see how significant he was in my academic life as well as in the lives of other epidemiologists who were trained by him. Besides being a brilliant mind, Dr Wenzel has the ability to bring people together to work towards a common cause. I have great admiration and friendship for him. However, I also had other very important mentors, such as Dr Michael Pfaller from microbiology at Iowa, who not only helped me with my research projects but also welcomed other Brazilian fellows from my university in Brazil (EPM/UNIFESP), such as Dr Antonio Carlos C. Pignatari (who later became a full professor), Dr Helio Sader, and Dr Ana Gales, who came in different subsequent years, following the next decades. We realized there was a need to improve microbiological diagnostics to enhance our epidemiological investigations, including outbreak investigations in Brazil, as well as to improve our understanding of epidemiology, knowing that we are quite dependent on them. Before coming to Charlottesville, VA, and Iowa, I had to take English courses. I took immersion courses in English in Atlanta before my move to Virginia. I was fortunate to stay with an American family, in the home of one of the CDC administrative directors, whose name I don’t recall.

In addition to my research at the university, I worked as an intensivist for two decades. During this time, I also received support and mentorship from Dr Elias Knobel, the head of the ICU at the time, who was known nationally and internationally in the field. This provided me with the opportunity to conduct research on critically ill patients, particularly in understanding the epidemiology of infections in this population.

## Other more specific contacts from that time that you would like to share?

Absolutely. Other contacts I made, such as Dr Mayhall, helped me recommend Dr Denise Cardo (now a former CDC’s director) to do her training in hospital epidemiology with Dr Glen Mayhall in Richmond, Virginia. Later, when Dr Mayhall moved to Memphis, she went there for further training and that’s where she met her husband, Dr Kenneth Leeper. Additionally, I met two doctors in the United States who spoke perfect Portuguese. One was Dr Lee Harrison, now a professor at the University of Pittsburgh, whom I met during a hospital infection training course in Charlottesville, Virginia. He later visited me at Hospital Israelita Albert Einstein in São Paulo, Brazil, and gave some lectures in excellent Portuguese. Another was Dr Louis Kirchhoff in Iowa City, Iowa, a polyglot who studies Chagas disease and speaks Portuguese very well. He is a prominent figure in this topic of tropical diseases that do not exist in the United States but are present in Brazil. We are fortunate to have Dr Kirchhoff studying this.

## You’ve combined careers in medical education, infection prevention, leadership, and eventually became chief medical officer. How did that unfold, and what inspired you to pursue these unique career paths?

When I started in the field of hospital infection upon my return to Brazil, there were no nurses working specifically in this area. We were the first to hire nurses specializing in infection control. Based on the experiences I gained during my training, I fought to maintain a ratio of one infection control nurse for every 250 beds. I tried to replicate in São Paulo what I learned in Virginia and Iowa, implementing the best practices of the time for preventing hospital infections in a place where it was still nonexistent, such as documenting hospital epidemiological active surveillance and changing practices like urinary collection bag. Imagine the underreporting of cases that existed and other potentially preventable infections. Basic things. Pneumonia was believed to occur, akin to spontaneous generation; there was no way to avoid it. I had to gradually change these concepts. It was an arduous task. I had to use the best of my medical relationship approach to implement everything I learned in practice.

I had the privilege of being the clinical director of a large hospital in Brazil, also widely recognized in Latin America. The Hospital Israelita Albert Einstein is one of the best hospitals according to US Newsweek (https://www.newsweek.com/rankings/worlds-best-hospitals-2024) – ranked 28th worldwide. At the “Einstein,” as we affectionately call it in Brazil, I was able to develop not only my skills as an infectious disease specialist and epidemiologist but also to bring a bit of science into private practice. During my tenure as clinical director, I also had the opportunity to participate in the implementation of protocols to improve patient safety at Einstein, and certainly, hospital infection made a significant contribution to achieving international certification from the Joint Commission International in 1999. The Hospital Israelita Albert Einstein, in São Paulo, Brazil, was the first hospital outside the United States to achieve this certification. And as I have mentioned before, Dr Elias Knobel supported me during my journey in the ICU as an intensivist until 1998.

## What are the rewards of training and mentoring Infectious Diseases (ID) fellows and junior faculty into stewardship and Infection Prevention (IP) careers? How has this benefited you personally?

The great reward is to train someone but also learn from them at the same time. Fortunately, I have friendships with all of them, and we continue to do so. One of these people was Dr Denise Cardo from the CDC. I trained other fellows in hospital epidemiology who came to us at EPM/UNIFESP. Great professionals who came from north to south of Brazil, and also fellows from Latin America such as Chile. Many of them are now professors at federal schools in my country. They also used the case-control methodology in their respective institutions, where I taught, and I know they are passing on these epidemiological teachings, of course now more updated, to their disciples as well. I have also mentored Dr Carmen Lucia Pessoa-Silva, who now holds a prominent position at the WHO. And considering that you always need to think about who will take your place, I have a good friend, Prof. Dr Eduardo Alexandrino S. de Medeiros, who did his master’s and doctorate under my guidance and has taken over all of my functions within the university (EPM/UNIFESP). He has further enhanced not only my research line but also for other healthcare professionals, including serving as a consultant to the Brazilian Ministry of Health.

## Which are your most meaningful publications to you? And why?

As I have mentioned before, the candidemia studies shaped my academic life in Brazil. Interestingly, at that time, I was inclined to study hot topics such as Methicillin-Resistant *Staphylococcus aureus* (MRSA), not *Candida*. Thrush in a child’s mouth. No one talked about *Candida* in Brazil. I have changed my mind, and hope that many others have too. Another publication that had a significant impact on me was one in which I had the privilege to participate with another friend whom I had mentored, Dr Lucieni Conterno (who also became a professor at a Brazilian state university, Universidade Estadual de Marilia). We published a paper in ICHE during the 1990s showing that *S. aureus* bacteremia has a high mortality rate, especially when the lung is the source of infection and when shock develops; resistance to methicillin may be another risk factor for poor outcomes, with an odds ratio of 4 times.^3^

## Finally, what do you do in your leisure time, and what would you recommend for future hospital epidemiologists?

I appreciate a lot of different things: contact with nature, biking, and lessons nature teaches us. Friendly people and reminiscing about good things from the past as this interview has provided me. For future hospital epidemiologists, I would recommend staying updated with the latest research and advancements in the field,^4^ networking with other professionals, and taking time for self-care to prevent burnout. Additionally, engaging in continuous learning through conferences, workshops, and mentorship opportunities can greatly benefit one’s career development. I would emphasize the importance of choosing your mentors wisely, as a good mentor can significantly shape your career. I believe that despite advancements in artificial intelligence, good mentors will always be irreplaceable. And if I can’t help anyone, at least not to hinder. That’s my philosophy.

## References

[1] Wey SB , Mori M , Pfaller MA , Woolson RF , Wenzel RP. Risk factors for hospital-acquired candidemia. a matched case-control study. Arch Intern Med 1989;149:2349–2353. 2802900

[2] Wey SB , Mori M , Pfaller MA , Woolson RF , Wenzel RP. Hospital-acquired candidemia. The attributable mortality and excess length of stay. Arch Intern Med 1988;148:2642–2645. 3196127 10.1001/archinte.148.12.2642

[3] Conterno LO , Wey SB , Castelo A. Risk factors for mortality in *Staphylococcus aureus* bacteremia. Infect Control Hosp Epidemiol 1998;19:32–37. 9475347 10.1086/647704

[4] Cotia ALF , Scorsato AP , Victor EDS , et al. Integration of an electronic hand hygiene auditing system with electronic health records using machine learning to predict hospital-acquired infection in a healthcare setting. Am J Infect Control. 2024;21:S0196-6553(24)00720-X. doi: 10.1016/j.ajic.2024.09.012. 39312966

